# The Morphology and Mechanical Properties of ESD Coatings before and after Laser Beam Machining

**DOI:** 10.3390/ma13102331

**Published:** 2020-05-19

**Authors:** Norbert Radek, Jacek Pietraszek, Aneta Gądek-Moszczak, Łukasz J. Orman, Agnieszka Szczotok

**Affiliations:** 1 Faculty of Mechatronics and Mechanical Engineering, Kielce University of Technology, Al. 1000-lecia P.P. 7, 25-314 Kielce, Poland; 2 Institute of Applied Informatics, Faculty of Mechanical Engineering, Cracow University of Technology, Al. Jana Pawła II 37, 31-864 Cracow, Poland; jacek.pietraszek@mech.pk.edu.pl (J.P.); gadek@mech.pk.edu.pl (A.G.-M.); 3 Faculty of Environmental, Geomatic and Energy Engineering, Kielce University of Technology, Al. 1000-lecia P.P. 7, 25-314 Kielce, Poland; orman@tu.kielce.pl; 4 Faculty of Materials Engineering, Silesian University of Technology, str. Krasinskiego 8, 40-019 Katowice, Poland; agnieszka.szczotok@polsl.pl

**Keywords:** ESD coating, laser beam machining, electrodes, morphology, mechanical properties

## Abstract

Electro-spark deposition (ESD) and laser beam machining (LBM) are the technologies using the concentrated energy flux. This paper deals with the issue of the impact of laser modification on the morphology and mechanical properties of carbide/copper coatings produced by electro-spark treatment. The coatings were applied to C45 carbon steel samples using the EIL-8A device. The following three types of electrodes made using the powder metallurgy (PM) hot pressing technique, from copper and tungsten carbide powders of different percentage compositions, were used for the coatings: 25% WC and 75% Cu; 50% WC and 50% Cu; and 75% WC and 25% Cu. Laser modification of the surface layers was performed with an Nd:YAG laser. The research focused on the analysis of the morphology of coatings applied by electro-spark technology before and after laser processing. The analysis of the morphology of electro-spark coatings revealed that the coatings had microcracks and pores. The laser beam machining of ESD coatings led to the homogenization of chemical composition, fragmentation of the structure, and elimination of microcracks. In addition, measurements of porosity, microhardness, adhesion, and analysis of XRD phase composition of the electro-spark coatings were performed. Laser processing proved to have a positive effect on improving the adhesion of coatings and reducing their porosity. This paper also presents a simulation model of heat transfer processes for the case of laser radiation impact on a WC-Cu coating. The developed numerical model, describing the influence of laser treatment on the distribution of temperature fields in the heated material (at a given depth) is of significant importance in the development of treatment technologies. Laser-modified ESD coatings perform anti-wear and protective functions, which enable their potential application in means of transport such as rolling stock.

## 1. Introduction

The development of manufacturing enterprises and their ability to compete in the market can mainly be characterized by the degree of innovative activity in relation to the production methods used. Effective introduction of new products to the market can be realized, from an economic point of view, only if the optimization options of both the product and production methods are recognized and analyzed soon enough. However, in many cases, technical capabilities derived from the traditional approach to the optimization of the manufacturing processes are insufficient, mainly when the considered materials are characterized by poor workability or cannot be manufactured using methods known to date.

In the era of globalization, the competitiveness of products is achieved by improving manufacturing processes by focusing on new technologies. The fact that the introduction of new products is usually accompanied by the use of new high-strength materials, leads to a significant increase in the demand for unconventional, high-performance machining processes. In addition to the optimization of already known and used processes, the effects are achieved by consistent implementation of new technologies. These include beam technologies using concentrated energy flux [1,2,3].

An important part of experimental research related to the industrial application is the continuous measurement and assessment of the geometric structure of the surface of machine and tool elements [4,5]. For the formation of desirable surface features, the growing role of technology with a concentrated energy stream is observed. These technologies are mainly used for machining elements made of difficult-to-cut construction materials, as well as for the production of elements of very complex shapes. Their production using traditional methods would be very labor intensive and time consuming.

A characteristic feature of the beam technologies is that the treatment of materials occurs as a result of the impact of the concentrated energy flux. By controlling the energy flux, the selected areas of the surface can undergo the treatment process. The diameter of the energy beam varies from a fraction of nanometers to several tens of centimeters. It depends on the basic physical phenomenon occurring in a given technology. The smallest field of impact is typical of the methods which use a stream of electrons or a laser beam. The energy streams, occurring here, are focused and controlled by optical or magnetic lens systems or mirrors, while the size of the spot in the focal point depends only on the wavelength of the electron or laser radiation.

By applying new engineering materials or protective coatings, it is possible to improve the functional properties of machine parts, so that they are resistant to corrosion, abrasion, and erosion, as well as possess high fatigue strength. The new materials, for example, alloy steels, are usually expensive, which is undesirable, because the higher the cost of the material, the higher the price of the finished product. However, if an element is to be subjected to high loads, then strength rather than cost is the primary factor.

There are several thermal and chemical processes for surface layer modification, such as nitriding, physical vapor deposition (PVD), chemical vapor deposition (CVD), electroplating, etc., which improve wear or corrosion resistance [6,7]. The cost of equipment, duration of the process, and negative impact on the natural environment are just some of the disadvantages of the methods mentioned. Electro-spark deposition (ESD) is an alternative that can eliminate some of these disadvantages. The ESD method is distinguished by the low cost of its application, a relatively short implementation time, and simple procedures for process implementation. Preparation of materials for this treatment consists of degreasing the surface of the substrate and the tip of the electrode with extraction gasoline [8].

The ESD treatment for the regeneration of the surface layer of jet turbines, chassis hydraulics, and tactical vehicles can be used to restore the functional properties of the elements at relatively low costs and within short periods of time [9,10]. Similarly, the broader use of ESD machining to create protective coatings for a wide range of automotive parts can significantly reduce costs in the automotive industry [11].

The impact of the parameters of electro-spark deposition on the coating properties is still the subject of research on the following: influence of a carbon content on coating properties [12], formation of ESD spot [13], ESD alloying [14,15], coating of biomedical implants [16], mass transfer modeling in ESD [17], titanium-based ESD [18,19], and analysis of the structure of electro-spark deposited coatings [20,21]. Rukanskis [22] has shown the advantages of the economical use of ESD coatings on metal surfaces. Many of the research papers concern the deposition of anti-wear electro-spark coatings on steel substrates to improve their operating properties [23,24,25,26,27].

The causes of defects in the electro-spark coating, such as cracks and delamination between the coating material and the substrate, are a research challenge which explains why it is not currently possible to create a coating using the ESD process that is free from the mentioned defects [28,29,30,31,32]. Good mechanical and tribological properties of the coatings can be achieved using hybrid technology which combines vacuum electro-spark alloying, cathodic arc evaporation, and magnetron sputtering [33,34].

However, it is possible to improve the quality of the coating using appropriate laser treatment [35,36,37].The coating structure is modified by the laser beam, which also significantly affects its mechanical and functional properties.

It should be expected that the advantages of laser machining of ESD coatings would include the following:

- Improvement of the smoothness of the coating surface;
- Reduction of coating porosity;
- Improvement of the adhesion of the coating to the substrate material;
- Increased wear resistance and galling;
- Reduction of adverse tensile stresses which will increase fatigue strength;
- Increased corrosion resistance.

The issue of manufacturing new materials based on WC and Cu and the search for their applications is of topical interest [38,39]. In their works, the authors presented WC-Cu sinters made using hot pressing. Their thermomechanical properties, for example, Archimedes’ density, thermal diffusivity, Vickers hardness, and elastic modulus were investigated. WC-Cu cermets were devised for thermal barriers between the plasma facing tungsten tiles and the copper-based heat sink in the first wall of nuclear fusion reactors [39].

The aim of this research was to achieve, through additional laser beam machining (LBM), an enhancement of the performance characteristics of ESD-made WC-Cu coatings that should lead to the reduction of structural defects and adhesion improvement. The rationale of selecting an investigation approach was to point out the most appropriate ESD electrode composition considering the subsequent processing phase, i.e., LBM. Regardless, the particular detailed results and obtained predictive model could be of practical interest for specific R&D and industrial applications, for example, sealing technologies, transport, etc. Further investigation should result in a more precise predictive model and reveal possible new technological factors of significant influence.

## 2. Materials and Methods

### 2.1. Materials

The electrodes used in the process of application of electro-spark coatings were produced from elementary mixtures of copper powders and ceramic powders of tungsten carbide. The WC powder was supplied by OMG, USA (Table 1), while the Cu powder was supplied by NEOMAT, Latvia (Table 1).

The substrate for the ESD coating were samples made of C45 steel (Table 2).

### 2.2. ESD Electrode Preparation

A two-factor three level mixture experimental design was selected as a base for the research with the following particular mixtures:

- 25% WC vs. 75% Cu (labeled WC25-Cu75);
- 50% WC vs. 50% Cu (labeled WC50-Cu50);
- 75% WC vs. 25% Cu (labeled WC75-Cu25).

**Figure 1:**
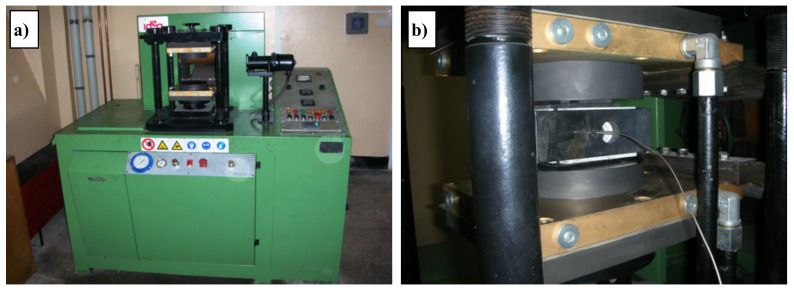
Idea furnace for the hot press process. (**a**) General view; (**b**) Matrix view with thermocouple installed.

Mixing of powders in appropriate proportions took place in a Turbula T2C mixer (ARTISAN TECHNOLOGY GROUP, Champaign, IL, USA). After filling the container to about 30% of its volume, the powders were mixed for 30 minutes at 66 rpm. The electrodes which were used to deposit coatings by means of the electro-spark deposition (ESD) method were produced using the powder metallurgy (PM) hot pressing route. A matrix was used to produce the electrodes, in which six 40 × 6 × H mm electrodes were pressed simultaneously. The height of the sinter (H) depended on the powder mass and sintering density. After using 12 g of powder weights, electrodes with a height of about 7 mm were obtained and such electrodes were used in experiment. All the mixtures were pressed hot (Figure 1) withstanding the powder for 3 minutes at 950 °C, at a pressure of 40 MPa, and therefore sinters were obtained with sufficient mechanical strength and porosity not exceeding 15%. The pressing temperature was measured using a thermocouple.

### 2.3. Electro-Spark Deposition

The equipment used for electro-spark deposition was the EIL-8A device (TRIZ, Sumy, Ukraine). The following operating parameters of the generator were determined experimentally: 0.7 A, 230 V, and 150 μF capacitors.

### 2.4. Laser Beam Machining

The laser treatment of the WC-Cu coatings was carried out with a Nd:YAG laser, model BLS 720 (*LASERTECHNIK* GMBH, Düren, Germany) with the following parameters: diameter of the laser beam d = 0.8 mm, laser power *P* = 60 W, scan speed *v* = 240 mm/min, pulse duration *t_i_* = 0.45 ms, frequency *f* = 55 Hz, and laser beam stroke *L* = 0.5 mm.

### 2.5. Morphology Testing

The aim of the analysis of the microstructure of the surface layer was to assess the cohesion of the superimposed surface layer with the substrate by means of the electro-spark method and the influence of laser processing parameters on the structure of the surface layer and substrate material.

During the process of the electro-spark layer application, the electrode components which acted as a binder were transformed into a liquid state. In the case of the layers studied, copper powders became liquid, which allowed for effective deposition of the layer together with tungsten carbide powders (WC). In order to determine the consistency of the deposited surface layer with the substrate material, as well as the homogeneity of the distribution of tungsten carbide powders, microscopic investigations were performed using light microscopy (Nikon Eclipse E400, Champaign, IL, USA) and scanning electron microscopy (Joel type JSM-5400, Peabody, MA, USA).

The evaluation of the structure of the materials tested was carried out on the specimens made in the plane perpendicular to the superimposed surface layer, which made it possible to assess the consistency of the layer with the substrate and to measure its thickness.

### 2.6. XRD Studies of Phase Composition

The phase composition of the coatings under investigation was analyzed by X-ray diffraction method using a Philips PW 1830 device. A filtered Kα of Cu anode lamp supplied with 40 kV at 30 mA current was used. The investigations were performed for angle 2θ in the range from 30° to 120° and scanning speed 0.05°/3 s.

### 2.7. Porosity Measurement

There are several ways to characterize the pore space. One is an imaging technique in which two-dimensional (2D) pore space images are routinely available.

The porosity of the WC-Cu layers was evaluated on the basis of a quantitative analysis of the images taken with a scanning electron microscope (SEM) Philips XL30/LaB_6_. The SEM was equipped with SIS software which was used to carry out the evaluation. 

The quantitative analysis of the pores was determined in accordance with the Cavalieri–Hacquert principle. According to the principle a measure of the analyzed porosity can be as follows:

- the volume fraction of the pores (the ratio of the total volume of the pores to the total volume of the analyzed layer);
- the area fraction of the pores (the ratio of the total area of the pores to the total area of the analyzed layer);
- the fraction of the pores on the length of the control line segment (the ratio of the total chords length crossing the pores to the total length of the control line segment on the surface of the specimen).

The porosity obtained with these three methods were of equal value.

### 2.8. Microhardness and Adhesion Tests

The measurements of microhardness were performed using the Vickers method with a Microtech MX3 tester under the load of 0.4 N, acting for 15 s. The indentations were made in perpendicular sections in the following three zones: the coating (before and after laser processing), the heat-affected zone, and the substrate. Each sample was tested ten times to generate the average values.

The adhesion measurements of the WC-Cu coatings before and after laser treatment were made with a scratch test. A Revetest Xpress Scratch Tester by the Swiss company CSM Instruments was used. The following scratch test parameters were applied:

- constant load of 20 N;
- table feed rate of 1200 μm/min;
- scratch length of 5000 μm;
- Rockwell diamond cone with rounded tip radius of 200 μm.

The scratch test involved the controlled scratching of the sample surface with a diamond indenter under varying loads. Under the impact of a certain, precisely defined load (force), called critical, the scratched coating (coating system) was damaged. Additionally, the scratch tester measured the normal force acting on the sample surface, friction force, and penetration depth. Automated microscopic observations also enabled the analysis of scratch lines along the entire length of the applied force. This observation made it possible to “identify” critical points during the test. The critical load was determined using an optical microscope. The depth of the “scratched” area (its linear dimensions) was strictly dependent on the given test parameters.

The smallest normal force causing a loss of adhesion of the coating (coating system) to the substrate is called the critical force F_cr_ and is considered to be a measure of adhesion.

The critical force value was evaluated using friction force changes and microscopic observations (optical microscope built into the Revetest Xpress Scratch Tester).

## 3. Experimental Results and Discussions

### 3.1. Structure Analysis Using Light Microscopy

The analysis of the microstructure of the surface layer included the analysis of the layer’s consistency with the substrate material and, in the case of the analysis of samples after laser treatment, the heat-affected zone (HAZ). In order to reveal elements of the granular structure, the surfaces of the samples were subjected to etching with Nital 7% reagent and exposure time of 30 s.

The observations of the images from the light microscopy allowed for qualitative and quantitative analysis of the surface layer and intermediate layer over a much larger area than would have been possible with scanning electron microscopy. The qualitative analysis assessed the cohesion of the applied layer with the substrate material before and after laser treatment, and analysis of the influence of laser treatment on the structural changes in the area of the surface layer and heat-affected zone.

**Figure 2:**
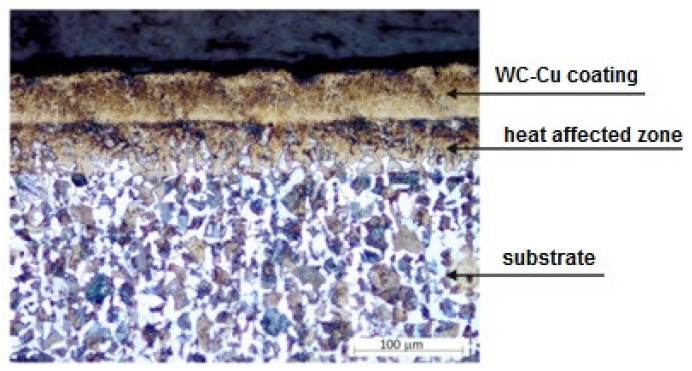
Sample cross-section with WC25-Cu75 coating after laser beam machining (LBM).

The quantitative analysis included measurement of the surface layer thickness before and after laser treatment and measurement of the heat-affected zone thickness. In the images obtained from the light microscope, the following individual zones of the examined sections can be clearly distinguished: WC-Cu coating, heat-affected zones, and substrate material (Figure 2).

On the basis of the observation of the cross-sections of all the samples after laser treatment we concluded that the surface layer after laser treatment showed very good cohesion with the substrate material. There were no material defects such as pores or cracks found. As a result of the laser treatment, the surface layer became homogenous. In the area of the heat-affected zone, a fine-grained and coniferous structure was observed as a result of phase transformations under the influence of laser surface treatment. In order to verify the influence of laser surface treatment parameters on the thickness of the surface layer and HAZ thickness, they were investigated. Measurements were carried out on cross-sectional images at ×200 magnification using interactive tools in the Aphelion 4.3 image analysis software.

The thickness of the applied WC25-Cu75 coating ranged from 36 to 68 µm and the WC50-Cu50 coating from 40 to 70 µm. The thickness of the WC75-Cu25 coating was 38 to 65 µm. The average thickness of the surface layer for the tested samples after LBM on the basis of the measurements was from 42 to 75 µm. The average thickness of the heat-affected zone was in the range of 34 to 51 µm.

### 3.2. SEM Analysis with EDS

The studies using electron microscopy were aimed at evaluating the morphology of the surface layer structure before and after laser treatment.

**Figure 3:**
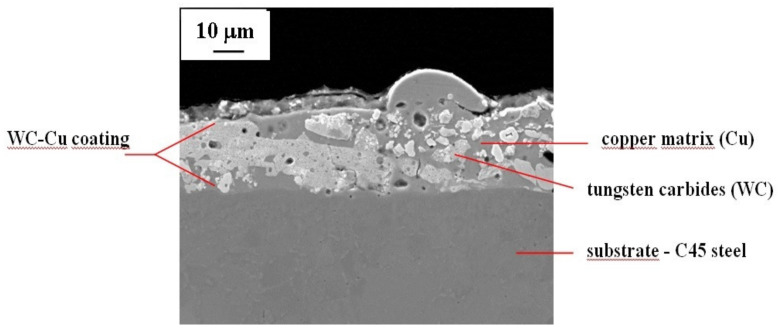
WC50-Cu50 coating components.

On the basis of metallographic images of the samples with the applied surface layer before laser treatment, it was possible to determine its individual components (Figure 3) as follows: copper matrix (Cu), tungsten carbide (WC), and substrate material.

As can be seen in the cross-sectional image of the sample with the electro-spark WC-Cu coating, it has a very good cohesion with the substrate and the boundary between the coating and the substrate material is clear. Tungsten carbides are quite homogeneously distributed in the copper matrix. Numerous pores and cracks are also visible in the cross-section of the coating.

**Figure 4:**
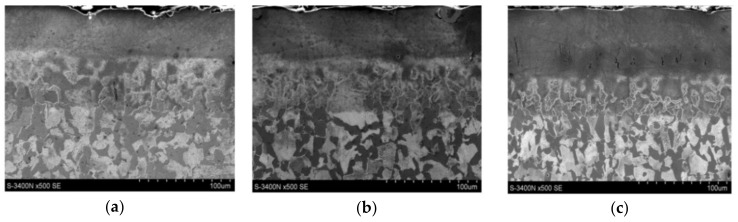
SEM images of the cross-sections of tested samples with a surface layer applied after laser treatment. (**a**) 25%WC-75%Cu; (**b**) 50%WC-50%Cu; (**c**) 75%WC-25%Cu.

The SEM observation (Figure 4) of the cross-sections of the WC-Cu coatings after laser treatment confirmed the results of the observation obtained using light microscopy. The average thickness of the laser-treated WC-Cu coatings ranged from 43 to 75 μm. Moreover, the heat-affected zone was in the range of 46 to 51 µm, and the content of carbon in the zone was higher.

For all samples, the following individual sample zones are clearly visible: coating, HAZ, and substrate material. The local heterogeneity of coating thicknesses due to laser processing is visible in the cross-sections, which is also reflected in the light microscopic images and the thickness measurements taken. Observation at ×500 magnification did not reveal any material defects such as pores, cracks, or local decohesion.

Analyses of structure and chemical composition were carried out to determine the microstructure, relative content of microstructural components, as well as the size and distribution of phase components of the coatings.

The chemical composition analysis using SEM with EDS detector was subjected to the surface of the WC-Cu coatings before and after laser modification. The results of the chemical composition analysis were presented in the form of a map of points, in which number and density correspond to the location and quantity of the element detected.

**Figure 5:**
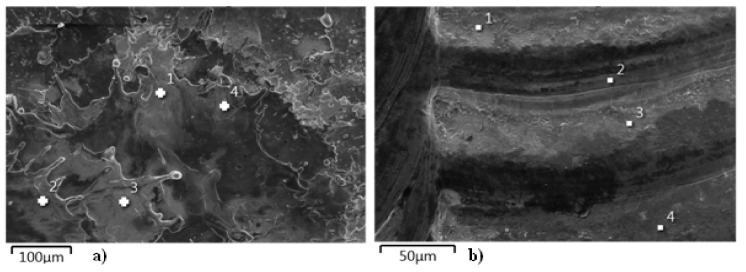
SEM image of the surface structure of the WC50-Cu50 coating. (**a**) Without laser beam modification; (**b**) After laser beam modification.

Figure 5 shows an example of the SEM image of the surface structure of the WC50-Cu50 coating before and after modification with a laser beam.

**Figure 6:**
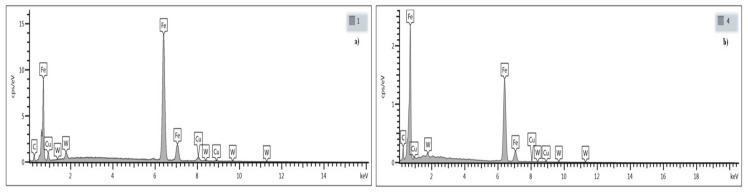
Results of EDS analysis of the chemical composition of the WC50-Cu50 coating. (**a**) Before LBM (point 1); (**b**) After LBM (point 4).

From the microstructure images shown in Figure 5, it can be concluded that the laser modification has significantly unified the state of the coating. As a result of the modification of the coating, the pores and microcracks are eliminated and the distribution of the elements (Figure 6b) is more uniform, which positively affects the modified surface.

The results of spectral analysis for selected points of the test surface are shown in Figure 6. The EDS analysis confirms the quantitative distribution of elements in the coating, where mainly iron, as well as copper, carbon, and tungsten can be found.

### 3.3. XRD Studies

After the analysis of the phase composition of the WC-Cu coatings, it turned out that the surface layer of the coatings consisted mainly of Feα, WC, and Cu and a small amount of W_2_C phase. The laser processing of the coatings did not cause major changes in their phase composition.

**Figure 7:**
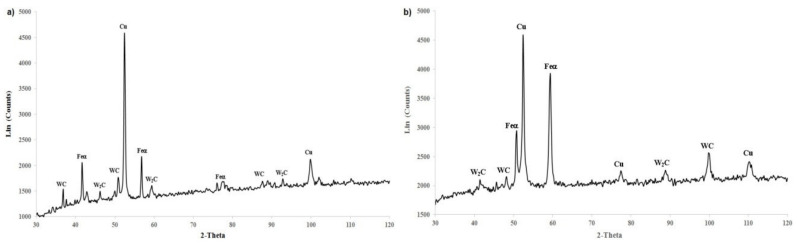
The XRD patterns of the WC25-Cu75 coating. (**a**) Without laser processing; (**b**) After laser processing.

Figure 7a,b show examples of the XRD patterns for the WC25-Cu75 coating before and after laser processing. When analyzing these images, it turns out that the surface layer of the coating without laser treatment consisted mainly of Cu (due to its 75% share in the coating), Feα, and a small amount of WC and W_2_C phases. The laser treatment did not cause the WC25-Cu75 coating to melt with the base material (Figure 7b). The surface layer of the WC25-Cu75 coating after laser treatment has a similar phase composition as that before treatment. An analysis of Figure 7a,b shows that the most intense peaks come from copper.

### 3.4. Porosity Measurement

**Figure 8:**
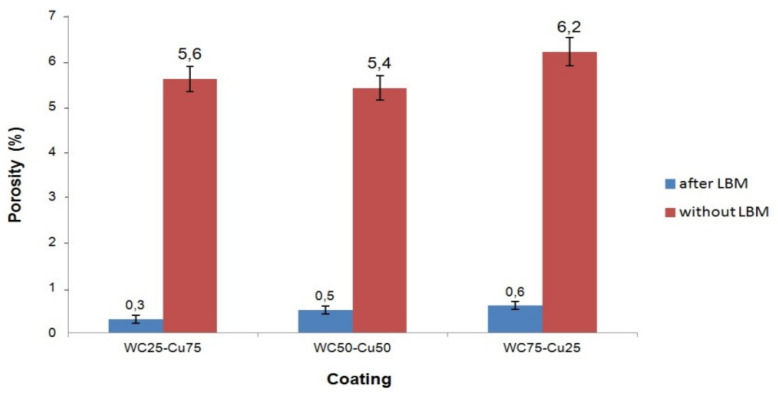
The mean value of the porosity of the layers.

The results of the porosity investigation of the WC-Cu layers before and after LBM application are presented in Figure 8. As shown in Figure 8, the applied laser treatment caused a reduction of the porosity of the WC-Cu layers.

The porosity of the layers after the LBM application was reduced over ten-fold as compared with the layers without such processing. The mean value of the porosity of the WC-Cu layers (computed on the basis of 10 measurements) was within a range between 5.4% and 6.2%, whereas after the LBM application, it was between 0.3% and 0.6%. The lowest value of the porosity occurred in the case of the WC50-Cu50 layer, and the highest value was noticed in the case of the WC75-Cu25 layer. After the laser treatment application, the WC25-Cu75 layer was characterized by the lowest porosity, and the WC75-Cu25 layer was characterized by the highest porosity. The differences between the porosity of the WC-Cu layers before and after laser modification could be the effect of the electro-spark and laser processing (the same parameters were applied for the all layers), as well as different chemical composition (in %wt.) of WC and Cu used for production of the electrodes for electro-spark processing.

The following conclusions can be assumed on the basis of the performed investigations: lower porosity of the WC-Cu layers exhibits profitable effect on their exploitation, i.e., increases their corrosion resistance, improves adhesion, and enhances microhardness.

### 3.5. Microhardness and Adhesion Tests

**Figure 9:**
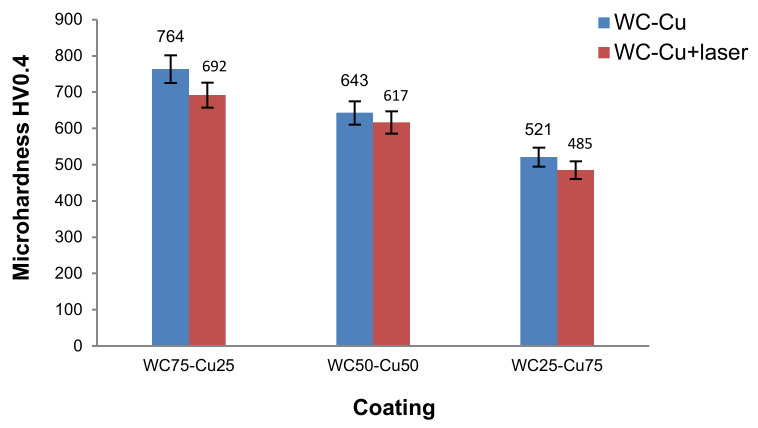
Results of microhardness measurements.

Electro-spark deposition caused changes in the microhardness of the treated material. Microhardness of the substrate before and after electro-spark deposition in all the coatings was on average ca. 278 HV0.4. The value of this parameter of the WC-Cu coatings in the heat-affected zone after the electro-spark treatment was ca. 58% to 63% higher than that of the substrate material. The application of the electro-spark deposited WC-Cu coatings led to a considerable increase in microhardness in relation to the substrate material. The WC-Cu coatings, depending on their composition, had different microhardness. The WC75-Cu25 coating proved to be the hardest. The average microhardness of the surface layer was ca. 764 HV0.4, which was over 2.5 times higher than that of the substrate material. The lowest average microhardness of 521 HV0.4 was observed in the case of the WC25-Cu75 coating. Laser beam machining slightly decreased microhardness of WC-Cu coatings by ca. 4% to 9% as compared with the coatings without such treatment (Figure 9).

A small decrease in microhardness of the coatings (after laser processing) can have a favorable impact on their elastic properties, which is of significance during operation under considerable loads, for example, in the case of drilling tools in the extractive industry or press elements used in building ceramics’ production. It could be caused by the dissolution of carbides. For further tests, the laser beam parameters have to be set in such a way that would be possible to eliminate the dissolution of carbides in the technologically formed surface layer.

Table 3 presents the critical force values from five measurements of a given sample, as well as the calculated average values and standard deviations.

**Figure 10:**
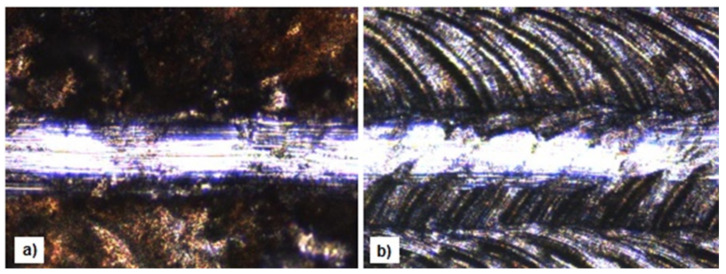
Surface images of the samples after the scratch test of the WC25-Cu75 coating (magnification x10). (**a**) Before laser processing; (**b**) After laser processing.

The measurements of adhesion carried out on the electro-spark coatings before and after laser irradiation showed different behaviors of the layers depending on the alloyed material and its subsequent laser treatment. The examples of microscopic images of the surface of the tested samples after conducting the scratch test are shown in Figure 10.

On the basis of the research data, it becomes evident that due to the laser treatment, it is possible to markedly improve adhesion of the WC-Cu coatings to the C45 steel substrate. Additionally, the results indicate that as the copper content increases, the coating’s adhesion to the substrate increases.

The WC25-Cu75 coating has the highest adhesion before and after laser processing. The average value of the critical force of the WC25-Cu75 coating calculated from five measurements was 10.12 N; after laser processing, it increased to 12.63 N. The LBM caused a 20% improvement in the adhesion of the WC25-Cu75 coating. Moreover, the low scatter of critical stylus loads indicates that the laser processing eliminates voids present at the coating/substrate interface. This was also confirmed by the porosity tests. In order to determine the values of critical forces causing damage to surface layers, it is advisable to use microscopic methods which would obtain more explicit results.

## 4. Numerical Simulations of the Impact of Laser Radiation on the Surface of Solids

### 4.1. Theoretical Framework

Solid-state energy transport (for isotropic bodies) is described by the equation:

(1) $$\rho c_{p}\frac{\partial T}{\partial t}+\nabla (k\nabla T)-q_{v}=0$$

This is the general equation of heat conduction in an isotropic body, which takes into account internal heat release, known as the Fourier–Kirchoff equation. Dividing the balance equation by *ρ*·*c_p_*, introducing thermal diffusivity *a*:

(2) $$a=\frac{k}{\rho c_{p}}$$

and the extension of the Nabla operator:

(3) $$\nabla (k\nabla T)=\frac{\partial }{\partial x}\left(k\frac{\partial T}{\partial x}\right)+\frac{\partial }{\partial y}\left(k\frac{\partial T}{\partial y}\right)+\frac{\partial }{\partial z}\left(k\frac{\partial T}{\partial z}\right)$$

leads to the final equation:

(4) $$\frac{\partial T}{\partial t}=a\nabla^{2}T+\frac{1}{\rho c_{p}}\frac{\partial k}{\partial T}\left[{\left(\frac{\partial T}{\partial x}\right)}^{2}+{\left(\frac{\partial T}{\partial y}\right)}^{2}+{\left(\frac{\partial T}{\partial z}\right)}^{2}\right]+\frac{q_{v}}{\rho c_{p}}$$

For the constant thermal conductivity (*λ* = const) and the absence of internal heat sources (*q_v_* = 0), the equation takes the form of the Fourier differential equation:

(5) $$\frac{\partial T}{\partial t}=a\nabla^{2}T$$

and the Laplace differential equation, if heat conduction is steady (independent of time $\left(\frac{\partial T}{\partial t}=0\right)$):

(6) $$\nabla^{2}T=0$$

In order to obtain a solution of a differential heat conduction equation, it is necessary to determine the conditions of the solution and only then, is the considered problem formulated correctly. These conditions include:

- geometrical conditions, determining the shape and dimensions of the body;
- physical conditions, determining physical properties of the substance, from which the body is made;
- distribution of efficiency of internal heat sources in time and space;
- boundary conditions, including:
  - initial conditions which determine the temperature distribution at the beginning,
  - boundary conditions which determine the heat transfer conditions on the outer surfaces of the body.

### 4.2. Numerical Analysis of Heat Transfer during Laser Processing of the WC-Cu Coating

In the course of the experimental research, a number of numerical simulations were carried out regarding the determination of temperature fields when absorbing laser radiation by complex solids of different geometries.

The tests were conducted for laser impacts on structures at a stationary laser beam, at beam motion at different speeds, for different geometrical systems and different beam powers.

The sample is in the shape of a cuboid made of C45 carbon steel.

It is assumed that the sample has a 100 µm coating and its material is a mixture of tungsten carbide and copper in the ratio of 1:1 WC/Cu (50% WC and 50% Cu). The red area on the front side of the model represents the laser dot with a diameter of ϕ 3 mm.

The physical properties of the materials are shown in Table 4. The material data of the WC-Cu coating were determined as the arithmetic mean of the values of the individual parameters.

In order to conduct the numerical analyses, a finite element mesh was prepared. It consists of rectangular elements. Due to the special interest in heat distribution in the first layer (WC-Cu coating), it consists of 10 elements in thickness. The energy distribution in the laser spot was approximated as a rectangular distribution, and therefore simple solver settings of the FLUENT program could be used for carrying out the analyses, i.e., boundary condition with a defined heat flux. The back wall of the sample was considered as being subjected to convective heat exchange under standard conditions (300 K and 20 W/m^2^K). The other walls were treated as adiabatic walls.

The calculations were performed for the laser power of 60 W. The assumed diameter of the laser spot is 3 mm, which gives the surface area of 7.065 × 10^−6^ m^2^. This means that the penetrating heat flux (for 60 W laser power) according to Equation (7) is 8.5 × 10^6^ W/m^2^.

(7) $$q=\frac{P}{A}$$

where *P* is the laser beam power and *A* is the surface area of the laser spot.

The calculations were carried out in a transient state with a time step of 1 ms. Despite the fact that the experiment used a Nd:YAG laser with an impulse operation mode (pulse duration t_i_ = 0.45 ms), the numerical analysis was conducted with the assumption of a continuous operation mode.

**Figure 11:**
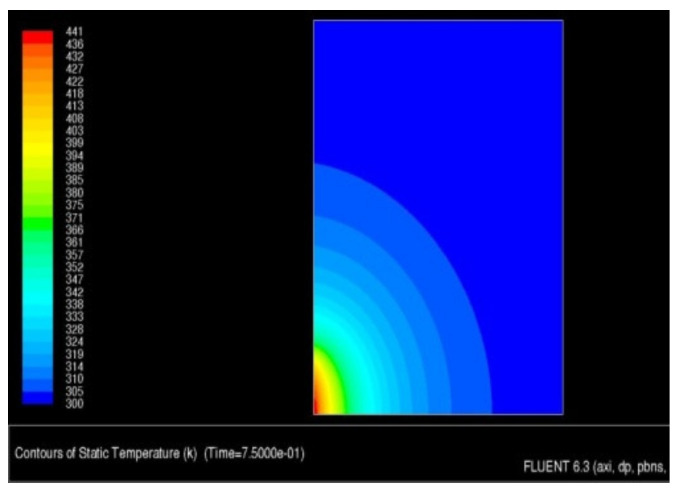
Temperature distribution in the sample after 75 ms.

The beginning of melting of the WC-Cu coating or the appearance of a steady-state heat conduction (whichever comes first) was assumed to be the end of the calculation time. The steady-state analysis showed an unacceptable increase in temperature in the sample, which could indicate insufficient heat dissipation. To eliminate this effect, it was decided to apply convective heat exchange to all external walls. The copper melting point was only reached after ca. 225 s. Therefore, the detailed results are presented for the time range of 0–1 s. The temperature distribution in the sample after 75 ms is shown in Figure 11.

**Figure 12:**
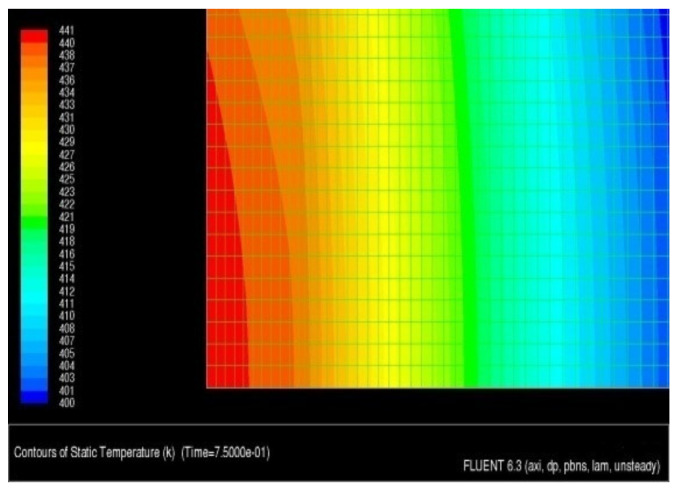
Temperature distribution in the technological surface layer.

**Figure 13:**
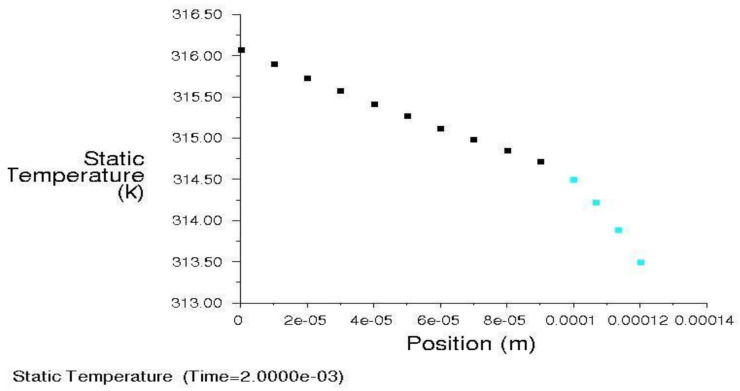
Temperature distribution in the technological surface layer.

As a result of the simulation, an animation was obtained that showed the heat transfer to the sample in the time range from 0 to 100 ms. The temperature distribution in the surface and transition layers is presented by a thermogram of the temperature field distribution (Figure 12) and the animation "dump" graph (Figure 13). As shown in Figure 13, the temperature limit of 441 K is located at a depth of about 45–50 µm.

The conducted analysis shows that the given laser power at a given spot size causes a very small increase in material temperature, i.e., approximately 150 K. In order to increase this, it is recommended to reduce the laser spot or increase its power. For example, for a laser spot of d = 0.8 mm and power P = 60 W, the heat flux would be 119 MW/m^2^, and with the current flux of 8.5 MW/m^2^, gives an increase of over 14 times.

## 5. Conclusions

On the basis of the obtained test results and their interpretation, the following conclusions can be drawn:

1. The surface of C45 carbon steel can be modified by means of electro-spark deposition using WC-Cu electrodes composed of various proportions of its two main constituents.
2. Laser beam machining significantly and favorably affects some properties of the ESD coating, but the degree of this effect strongly depends on the composition of the coating.
3. Laser treatment strongly (over ten-fold) reduces the porosity of the coating layer.
4. Laser processing slightly (on the statistical significance edge) reduces microhardness (approximately 4% to 9%).
5. Laser radiation causes an improvement in the functional properties of the electro-spark deposited WC-Cu coatings, i.e., they exhibit higher resistance to adhesion. Laser treatment increases the adhesion of WC-Cu coatings, on average, from approximately 19% to 27%.
6. The surface layer of the WC-Cu coatings before and after LBM consists of mainly Feα, WC, and Cu, as well as a small amount of W_2_C phase.
7. As a result of laser remelting of electro-spark coatings and subsequent solidification, the chemical composition of the coatings has changed. Laser irradiation of coatings resulted in the homogenization of the chemical composition, fragmentation of the structure, and elimination of microcracks.
8. As a result of the laser beam’s impact on a solid with a complex structure, the key to process control is the changing of the beam parameters. Nevertheless, the numerical simulation makes it possible to predict in a relatively simple and effective way the effects of modified laser radiation in a solid, depending on the material or geometry of the body and boundary conditions used. The basic element, when using the results of calculations, is the knowledge of the material data and the realization in physics of a given process adopted for the calculation of initial boundary conditions.
9. Further research should involve measurements of internal stresses and investigations into the erosion resistance tests of electro-spark coatings before and after laser beam machining.

## Figures and Tables

**Table 1:** Properties of the Cu and WC powders.

Powder	Species	Particle Size (µm)	Producer
WC	FL	~0.2 *	OMG (Cleveland, OH, USA)
Cu	Superfine	~0.04 *	NEOMAT (Salaspils, Latvia)

* measured using a Fisher Sub-Sieve Sizer.

**Table 2:** Chemical composition of C45 carbon steel.

Elements	C	Mn	Si	P	S
Content (%)	0.42–0.50	0.50–0.80	0.10–0.40	0.04	0.04

**Table 3:** Results of adhesion measurements of WC-Cu coatings.

Layer	F_cr_(N)					Average (N)	Standard Deviation (N)
	Measurement Set						
	1	2	3	4	5		
WC75-Cu25	7.74	6.34	7.21	6.26	7.83	7.10	0.79
WC75-Cu25 + laser	9.65	7.85	8.73	8.09	9.42	8.75	0.67
WC50-Cu50	8.67	7.93	9.12	8.34	7.26	8.24	0.60
WC50-Cu50 + laser	10.71	12.06	10.94	11.12	11.97	11.34	0.44
WC25-Cu75	9.37	10.13	10.87	10.43	9.06	10.12	0.72
WC25-Cu75 + laser	11.53	13.06	13.29	11.81	12.79	12.63	0.53

**Table 4:** Physical properties of the materials.

**Tungsten Carbide**				
**Temperature (K)**	**Density (kg/m^3^)**	**Heat Capacity (J/kgK)**	**Thermal Conductivity (W/mK)**	**Melting Point (K)**
273	15,800	171	84.02	3146
373	-	203	-	
573	-	231	-	
773	-	248	-	
**C45 Carbon Steel**				
**Temperature (K)**	**Density (kg/m^3^)**	**Heat Capacity (J/kgK)**	**Thermal Conductivity (W/mK)**	**Melting Point (K)**
273	7850	486	49.8	1813
473	-	519	-	
673	-	586	-	
**Copper**				
**Temperature (K)**	**Density (kg/m^3^)**	**Heat Capacity (J/kgK)**	**Thermal Conductivity (W/mK)**	**Melting point (K)**
273	8930	385	401	1353
300	-		398	
1000	-		357	
**WC-Cu (50% WC and 50% Cu)**				
**Temperature (K)**	**Density (kg/m^3^)**	**Heat Capacity (J/kgK)**	**Thermal Conductivity (W/mK)**	**Melting Point (K)**
273	12,365	278	242.51	2250
373	-	294	-	
573	-	308	-	
773	-	317	184.81	
